# Continuous Hemofiltration Reduces Mortality in Severe Acute Pancreatitis: A Meta-Analysis

**DOI:** 10.1155/2020/6474308

**Published:** 2020-06-29

**Authors:** Yulin Guo, Feng Cao, Chen Li, Huaxia Yang, Shaoyou Xia, Fei Li

**Affiliations:** ^1^Department of General Surgery, Xuanwu Hospital, Capital Medical University, Beijing 100053, China; ^2^Department of General Surgery, PLA Army General Hospital, Beijing 100853, China

## Abstract

**Background:**

Severe acute pancreatitis (SAP) is a deadly condition, with a mortality rate ranging from 15% to 30%. Recently, blood purification therapy has been adopted in administrating SAP patients. The present study aimed at evaluating the effect of continuous hemofiltration therapy for SAP.

**Methods:**

A systematic search of Cochrane Library, PubMed, and Embase was carried out until October 1^st^, 2019. Prospective studies comparing outcomes for SAP patients between continuous hemofiltration and standard therapy were enrolled.

**Results:**

Continuous hemofiltration therapy was associated with lower level of PACHE II score (MD = −1.49; 95% CI: −2.69 to −0.29, *P*=0.02), CRP (MD = −1.56 mg/L; 95% CI: −2.64 to −0.47, *P*=0.005), Cr (MD = −3.57 umol/L; 95% CI: −5.50 to −1.65, *P*=0.003), and Bun (MD = −3.63 mmol/L; 95% CI: −6.07 to −1.20, *P*=0.003) at 72 h after onset of treatment. Continuous hemofiltration therapy was associated with shorter length of abdominal pain relief time (MD = −1.82 hours; 95% CI: −2.93 to −0.71, *P*=0.001), lower surgery rate (OR = 0.15; 95% CI: 0.03 to 0.78, *P*=0.02), and mortality rate (OR = 0.54; 95% CI: 0.37 to 0.77, *P*=0.0007).

**Conclusions:**

continuous hemofiltration therapy could effectively alleviate SAP as early as 72 hours after onset of treatment, lowering the level of Bun, Cr, CRP, and APACHE II scores. Continuous hemofiltration therapy could confer SAP patients with lower mortality rates.

## 1. Introduction

The incidence of acute pancreatitis is 13 to 45/100,000, with about 20–30% of them presenting with severe acute pancreatitis (SAP) every year [1, 2]. SAP is a life-threatening condition characterized by local inflammation involving surrounding tissues and systemic inflammatory response syndrome (SIRS) which could contribute to multiple organ dysfunction syndrome (MODS). The mortality rate of SAP is ranging from 15% to 30%, irrespective of the advances in supportive care and technical development in therapy [2, 3].

The standard treatment of SAP consists of fasting, gastrointestinal decompression, parenteral nutritional, administration of somatostatin, and fluid restoration therapy. Recently, blood purification therapy has become more commonly used in administrating SAP patients. As cytokines and inflammatory products excessively released during the pathological process of SIRS in SAP are considered to be crucial mediators leading to MODS, continuous hemofiltration could remove these inflammatory mediators from the circulation by means of convective filtration, dispersion, and adsorption, blocking the pathological process and eliminating the cytokine cascade [4–6]. Despite some study opposing the benefits brought by the continuous hemofiltration for SAP, most studies have reported the effectiveness of continuous hemofiltration on SAP and its complications, but there are still some items such as abdominal pain relief, the cost of hospitalization, and the need for surgery that need to be further explored [7, 8]. The superiority of continuous hemofiltration for SAP needs to be well defined. The present study was carried out to determine the effect of continuous hemofiltration therapy for SAP, with strict study selection and more profound analyzed items.

## 2. Material and Methods

### 2.1. Literature Search Strategy

The present study was performed and reported according to the Preferred Reporting Items for Systematic Reviews and Meta-Analyses (PRISMA) guidelines with a PRISMA checklist and algorithm. Databases including Cochrane Library, PubMed, and EMBASE were searched to find potential studies that evaluate the effect of continuous hemofiltration therapy for patients with SAP (until October 1^st^, 2019). The search terms were as follows: pancreatitis, ((hemofiltration or haemofiltration) or Diahemofiltration), (((blood purification) or (blood replacement)) or CRRT) [continuous renal replacement therapy, CRRT]. These terms and their combinations were used in the process of searching. And the searching algorithm was as follows: ((((((((hemofiltration OR haemofiltration) OR Diahemofiltration) OR (blood purification)) OR (blood replacement)) OR CRRT)) AND pancreatitis) AND acute) AND severe. Moreover, to broaden the sources of potential articles, the “related articles” function was applied. The references of related articles were also traced with carefulness for potential articles on this topic. No language restriction was applied.

### 2.2. Study Selection

The inclusion criteria for the present study include the following: (1) SAP was diagnosed according to the Atlanta classification and its subsequent revised versions or the diagnostic criteria developed by the Chinese Medical Association. (2) Adult patients were prospectively grouped to receive continuous hemofiltration therapy or the standard treatment once the patient was diagnosed as SAP. (3) Studies reported at least one of the primary outcomes after continuous hemofiltration therapy. (4) The studies should be published as full-length articles. (5) Chinese studies should have an English abstract.

The exclusion criteria include the following: (1) Letters, case reports, case series, conferences, and reviews without original data were excluded. (2) SAP patients presented with acute renal failure at admission. (3) SAP patients presented with a history of chronic kidney diseases and need regular dialysis therapy before admission. (4) The study lacks a control group. (5) Retrospective study, in which grouping was made based on the treatment history but not the initial grouping plan, was excluded because of the improper design. For example, patients received continuous hemofiltration treatment according to whether there exists a complication but not the grouping plan [9]. (6) SAP patients in the control group received continuous hemofiltration therapy after the onset of treatment [7, 10]. (7) Apart from the continuous hemofiltration therapy, patients in the study group receive traditional Chinese medicine, plasma exchange, hemoperfusion, or other kinds of combination therapies [11, 12]. (8) Studies with small group size (less than 10 patients) were excluded [13]. (9) For studies involving an overlapped population, only the better-quality one could be included. 10) Studies lack baseline information, the necessary outcome of interests, or English abstracts were excluded.

EndNote X6 software was employed to perform the process of study searching and selection. Two authors independently scanned the titles and abstracts of the retrieved studies to determine potential studies. Then, the full texts of these studies were carefully assessed, in accordance with the inclusion and exclusion criteria. Disagreement happening during the selection process was discussed and resolved by participants from Beijing Union Medical College.

### 2.3. Data Collection and Quality Assessment

Data were extracted by two authors independently. General information extracted was as follows: author information, publication date (year), clinical characteristics, and research type. The outcomes of interest were outcomes after continuous hemofiltration therapy. The primary endpoints in the present study included APACHE II score, surgery rate after treatment, and mortality rate. These primary endpoints were adopted to assessing the effect of continuous hemofiltration on the remission and survival outcomes of SAP patients.

The Jadad scale and the Newcastle–Ottawa Scale (NOS) were applied to assess the methodological quality of randomized clinical trials (RCTs) and other prospective studies, respectively. The Jadad scale consists of three methodological items: randomization (0–2 points), blinding (0–2 points), and dropouts and withdrawals (0-1 point). RCT that achieve a Jadad score of 3 or more are of moderate to high quality. For NOS evaluation, three methodological items should be concerned: sample selection, design of the control and comparability, and outcome assessment. Studies that acquire a NOS score of six or more are of moderate to high quality. Disagreement happening during the selection process was discussed and resolved by participants from Beijing Union Medical College.

### 2.4. Statistical Analysis

Data were synthesized with Review Manager (The Cochrane Collaboration, Version 5.3, UK), and Adobe Photoshop software (Adobe Systems Software Ireland Ltd., Version CS5, USA) was used to create the artwork. Mean differences (MDs) and 95% confidence intervals (CIs) were calculated to analyze the continuous data. For dichotomous data, odds ratios (ORs) and 95% CIs were applied. When continuous data were reported as median with range, the mean and standard deviation were estimated using the method reported previously [14]. To evaluate the heterogeneity across studies, the I^2^ test was applied. If *P* > 0.1 or I^2^ value <50%, the assumption of study homogeneity was accepted and a fixed effect model was used. Otherwise, a random-effect model was used. Sensitivity analysis was performed to assess the strength and reliability of results by excluding one study in turn. Subgroup analysis was conducted regarding the patterns of hemofiltration. When data of interest under hemofiltration was reported by less than two of the included studies, the subgroup analysis was not conducted. Publication bias was assessed by funnel plot. Besides, Begg's and Egger's tests were employed to aid in detecting publication bias with STATA software (Version 10.0, STATA Corporation, Texas, USA). A *P* value <0.05 indicates statistical significance.

## 3. Results

### 3.1. Study Characteristics and Quality Assessment

**Figure 1:**
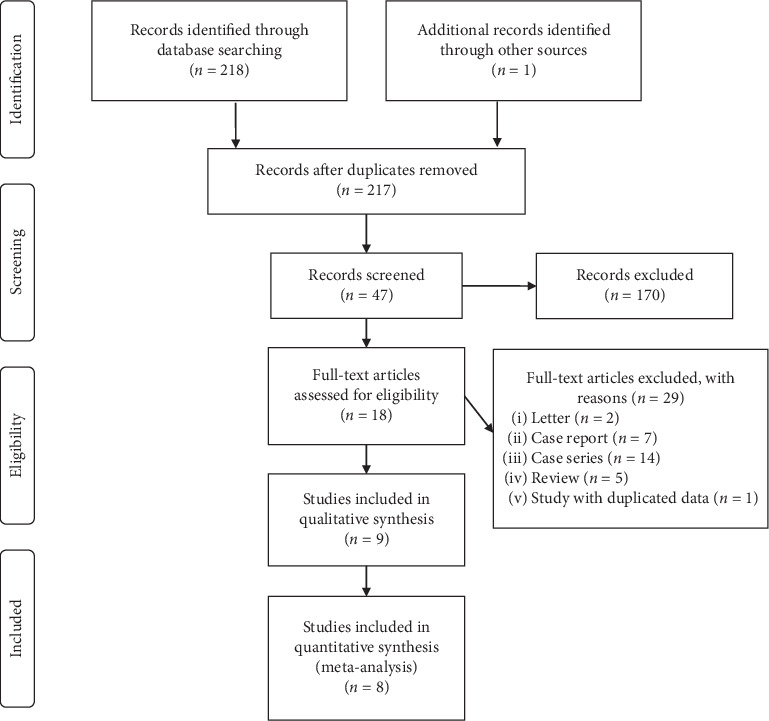
Flow diagram of literature search and selection.

We obtained 219 studies according to the systemic search, and 2 repetitive studies were removed. After reading titles and abstracts, 170 unrelated studies, 2 letters, 7 case reports, 14 case series, 5 reviews, and 1 study with duplicated data were removed. Then, there remained 18 studies, and full texts of these studies were carefully checked. Finally, 9 studies met the inclusion criteria for qualitative synthesis (Table 1) (Figure 1) [15–23]. In quantitative synthesis, 8 studies were included (Table 1) (Figure 1) [15–22]. A total of 470 patients were eligible in quantitative synthesis, with 242 patients receiving continuous hemofiltration therapy and 228 controls. General information including the characteristics and demographics of all included studies is shown in Table 1. The quality of most included RCTs except Wang et al.'s [21] study was relatively low. However, all the 2 prospective studies scored eight stars, indicating high quality (Table 1).

### 3.2. The Pooled Results of Items regarding Biochemical Tests and Symptoms

Data on CRP at 72 h after treatment were reported by four studies, involving 202 patients. The level of CRP was significantly decreased by the continuous hemofiltration therapy at 72 h after the onset of treatment compared with standard therapy (MD = −1.56 mg/L; 95% CI: −2.64 to −0.47, *P*=0.005; I^2^ = 91%, *P* < 0.00001 for heterogeneity) (Table 2) [16, 19, 20, 22]. Data on ALT at 72 h after treatment were available in three studies, involving 164 patients. The pooled results of ALT at 72 h after the treatment showed no statistical difference (MD = −0.35U/L; 95% CI: −0.83 to 0.13, *P*=0.16; I^2^ = 57%, *P*=0.10 for heterogeneity) (Table 2) [18, 20, 22]. As for items considering the kidney function, four studies reported Cr at 72 h after treatment, involving 201 patients. The level of Cr was significantly decreased by the continuous hemofiltration therapy at 72 h after the onset of treatment compared with standard therapy (MD = −4.96 umol/L; 95% CI: −7.77 to −2.15, *P*=0.0005; I^2^ = 98%, *P* < 0.00001 for heterogeneity) (Table 2) [16, 18, 20, 22]. Data on Bun at 72 h after treatment were reported by four studies, including 201 patients. The pooled results also showed a significantly lower level of Bun in the continuous hemofiltration group at 72 h after the onset of therapy (MD = −3.63 mmol/L; 95% CI: −6.07 to −1.20, *P*=0.003; I^2^ = 97%, *P* < 0.00001 for heterogeneity) (Table 2) [16, 18, 20, 22]. The abdominal pain relief time for SAP patients was reported by three studies, involving 185 patients. The pooled results showed a significantly shorter length of abdominal pain relief time for patients receiving continuous hemofiltration therapy (MD = −1.82 hours; 95% CI: −2.93 to −0.71, *P*=0.001; I^2^ = 86%, *P*=0.0006 for heterogeneity) (Table 2) [15, 21, 22].

### 3.3. The Pooled Results of Items regarding Primary Endpoints and Hospitalization

Three studies reported the APACHE II (Acute Physiology and Chronic Health Evaluation II) score at 24 h after the onset of treatment, involving 249 patients. Difference in the APACHE II score at 24 h after treatment between the groups was not significant (MD = −.41; 95% CI: −5.61 to 0.34, *P*=0.09; I^2^ = 88%, *P*=0.0002 for heterogeneity) (Table 2) [17, 19, 21]. APACHE II score at 72 h after the treatment was reported by five studies, with 350 patients. The pooled results showed that continuous hemofiltration therapy could significantly decrease the APACHE II score of SAP patients at 72* *h after treatment compared with standard treatment (MD = −1.80; 95% CI: −3.15 to −0.44, *P*=0.009; I^2^ = 96%, *P* < 0.00001 for heterogeneity) (Table 2) [18–22]. When the severity of SAP got worse despite the routine or continuous hemofiltration therapy, these patients would need surgical intervention. Data on surgery rate were shown in three studies, involving 121 patients. The pooled results showed surgery rate for patients in the continuous hemofiltration group was significantly lower (OR = 0.15; 95% CI: 0.03 to 0.78, *P*=0.02; I^2^ = 64%, *P*=0.06 for heterogeneity) (Table 2) [15, 19, 22]. The overall mortality rates after the treatment were reported by five studies, with 323 patients. The pooled result showed significant lower mortality rates for patients receiving continuous hemofiltration therapy (OR = 0.57; 95% CI: 0.37 to 0.85, *P*=0.007; I^2^ = 0, *P*=0.98 for heterogeneity) (Table 2) [16, 18, 19, 21, 22]. Both length of hospital stay [15, 16, 19, 21, 22] and the cost of hospitalization [15, 21, 22] were comparable between the therapies (Table 2).

### 3.4. Sensitivity Analysis

As significant heterogeneity was observed, sensitivity analysis was conducted in the following items: APACHE II score at 24 h after treatment, APACHE II score at 72 h after treatment, CRP at 72 h after treatment, ALT at 72 h after treatment, and Cr at 72 h after treatment, Bun at 72 h after treatment, the abdominal pain relief time, surgery rate, and mortality.

For APACHE II score at 24 h after treatment, heterogeneity remained high when carrying out the sensitivity analysis. After excluding Wang et al.'s study [21], APACHE II score at 24 h became significantly lower for patients receiving continuous hemofiltration therapy (MD = −3.67; 95% CI: −6.40 to −0.94, *P*=0.008; I^2^ = 65%, *P*=0.09 for heterogeneity). For APACHE II score at 72 h after treatment, sensitivity analysis was carried out with no significant change in results. The heterogeneity of the APACHE II score at 72 h after treatment disappeared after excluding the Wang et al.'s study [21]. As for CRP, ALT, Cr, and Bun at 72 h after the treatment, high heterogeneity existed consistently with no significant change in these results. For abdominal pain relief time, heterogeneity disappeared after the removal of Abulimiti et al.'s study [22], but no significant change in results was observed. As for the surgery rate, the heterogeneity disappeared after the removal of Abulimiti et al.'s study [22]. After the removal of Mao et al.'s study, surgery rate became comparable between the groups (OR = 0.12; 95% CI: 0.01 to 1.35, *P*=0.09; I^2^ = 82%, *P*=0.02 for heterogeneity) [15]. For mortality, the significance remained consistent with no significant heterogeneity in these results.

### 3.5. Publication Bias

**Figure 2:**
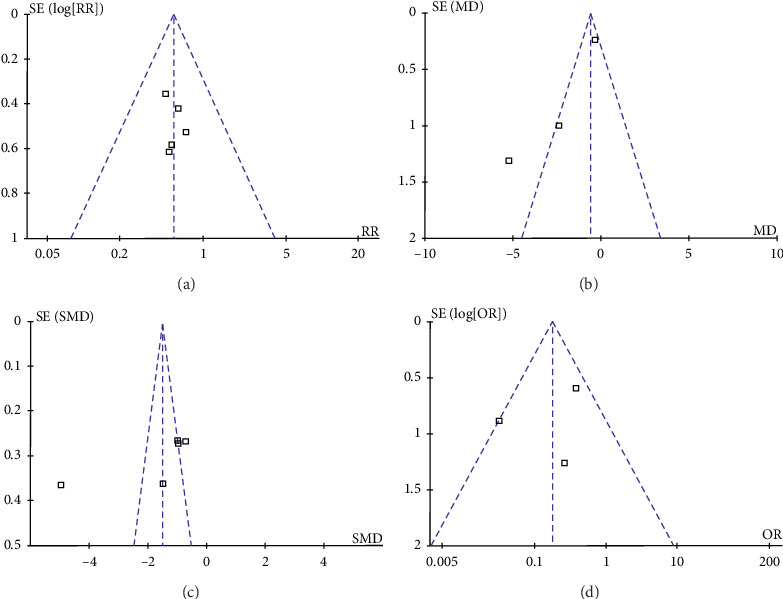
Funnel plot for pooled results. (a) Funnel plot of mortality rate after the treatment. (b) Funnel plot of APACHE II score at 24 h after the onset of treatment. (c) Funnel plot of APACHE II score at 72 h after the onset of treatment. (d) Funnel plot of surgery rate after the treatment.

The funnel plot on mortality showed no publication bias without studies in significant areas (Figure 2(a)). As the results of the APACHE II score at 24 h after treatment, APACHE II score at 72 h after treatment, and surgery rate were not stable during sensitivity analysis, both Begg's and Egger's tests were performed. The funnel plot on the APACHE II score at 24 h after the onset of treatment showed one study lied in the significant areas, indicating an existence of publication bias (Figure 2(b)) [17, 19, 21]. However, no publication bias was found by Begg's and Egger's tests (Begg's tests, *P*=0.296; Egger's tests, *P*=0.158). The funnel plot on APACHE II scores at 72 h after treatment showed two studies analyzed were in the significant areas, which indicated the existence of publication bias (Figure 2(c)) [18–22]. However, there was also no publication bias found by both Begg's and Egger's tests regarding APACHE II score at 72 h after treatment (Begg's tests, *P*=0.806; Egger's tests, *P*=0.542). As for the surgery rate, the funnel plot showed one study analyzed lied on the boundary line (Figure 2(d)) [19]. But no publication bias was found by Begg's and Egger's tests (Begg's tests, *P*=1.000; Egger's tests, *P*=0.713). Although no publication bias was detected by Begg's and Egger's tests on the above items, these results may be limited by the small size of the studies included.

### 3.6. Subgroup Analysis

Subgroup analysis regarding the hemofiltration mode was conducted. Items of interest consisting of data from more than two of the included studies were analyzed. Thus, pooled analysis of these items regarding HVHF treatment was conducted. APACHE II score at 72 h after the HVHF treatment was reported by three studies, with 161 patients. The pooled results showed that HVHF could significantly decrease the APACHE II score of SAP patients at 72 h after treatment compared with standard treatment (MD = −0.97; 95% CI: −1.30, −0.64, *P* < 0.00001; I^2^ = 30%, *P*=0.24 for heterogeneity) (Table 3) [18, 19, 22]. Data on ALT at 72 h after HVHF treatment were available in two studies, involving 100 patients. The pooled results showed that HVHF could significantly decrease the level of ALT at 72 h after the treatment (MD = −0.56 U/L; 95% CI: −0.96, −0.15, *P*=0.007; I^2^ = 51%, *P*=0.15 for heterogeneity) (Table 3) [18, 22]. However, there was no significant difference found between the HVHF and standard treatment considering the level of CRP, Cr, and Bun at 72 h after treatment (Table 3). Data on surgery rate were shown in two studies, involving 101 patients. The pooled results showed surgery rate for patients receiving HVHF treatment was significantly lower than that of patients who received the standard treatment (OR = 0.17; 95% CI: 0.07, 0.42, *P*=0.0001; I^2^ = 82%, *P*=0.02 for heterogeneity) (Table 3) [19, 22]. The mortality rates after the treatment were reported by three studies, with 161 patients. The pooled result showed a significant lower mortality rates for patients receiving HVHF treatment than that of patients who received the standard treatment (OR = 0.55; 95% CI: 0.33, 0.92, *P*=0.02; I^2^ = 0, *P*=0.82 for heterogeneity) (Table 3) [18, 19, 22].

## 4. Discussion

The present study conducted a meta-analysis comparing the efficacy between continuous hemofiltration and standard therapy in patients with SAP. The present study showed that continuous hemofiltration treatment in this patient group was associated with a significant reduction in the level of APACHE II score, CRP, Bun, and Cr at 72 h after the onset of treatment as well as the abdominal pain relief time, contributing to a significant reduction in the incidence of surgery rate and mortality.

As a severe inflammatory status, SAP induces excessive leukocyte activation and migration of neutrophils to the inflamed area contributing to a consequent release of inflammatory mediators, which results in an uncontrolled pathogenic progression of pancreatic infection to necrosis and SIRS [24, 25]. Thus, treatment strategies aimed at interrupting this process could be effective. Blood purification therapy including hemofiltration, hemodiafiltration, hemodialysis, and other modalities, was traditionally used for AKI (acute kidney injury) and sepsis. Blood purification could nonspecifically remove the inflammatory mediators with moderate molecular weights such as tumour necrosis factor-*α*, interleukin (IL)-1*β*, IL-6, and IL-8, which can activate and lead to progressively “waterfall-like” chain reaction playing an important role in the pathogenesis of SIRS [26, 27]. Moreover, studies also concluded that continuous hemofiltration could decreases the systemic overflow of inflammatory mediators, which helps restoring the balance between body proinflammatory system and anti-inflammatory system [28, 29]. Because the uncontrolled cytokines and inflammatory mediators excessively released during the pathological process of SIRS in SAP are considered to be crucial mediators leading to MODS and even death, the above effect of continuous hemofiltration could be effective on the treatment of SAP. Recently, continuous hemofiltration has been widely adopted in severe inflammatory status such as SAP, and a previous study had confirmed the effect of continuous hemofiltration on decreasing the level of inflammatory mediators for SAP patients [4].

In the present study, the patients treated with continuous hemofiltration, administered in addition to routine treatment, showed a significantly lower level of Cr and Bun compared to those in the control group as early as 72 hours after the onset of treatment. These results confirmed the effect of continuous hemofiltration on kidney function and are similar to the results after the CVVH (continuous venovenous hemofiltration) treatment from a retrospective study conducted by Chen et al. [30]. The effect of continuous hemofiltration on SAP could be proved by the significant reduction in CRP and APACHE II scores at 72 h after the initiating of treatment in the present study, for CRP is the marker of the inflammatory status and APACHE II score is the indicator of acute physiological status. The reduced levels of CRP and APACHE II scores at 72 h after the treatment highlighted the effect of continuous hemofiltration on clearing inflammatory mediators and improving immune function, which thereby decreased the mortality rate of SAP patients in the present study [5]. This result is in line with the study conducted by Miao et al. that reported the APACHE II score of SAP patient who had received a 72 h continuous hemofiltration was significantly lower than the patient under routine treatment [31]. In the retrospective study conducted by Wu et al., both CRP and APACHE II score for AP patients from the CRRT group were significantly lower than those of patients from the control group after treatment. Moreover, the mortality rate for the CRRT group was lower, though the difference did not reach significance [9]. In the present study, continuous hemofiltration contributed to a significantly lower rate for SAP patients who turned to surgical intervention after the treatment, which may further confirm that continuous hemofiltration might alleviate SAP and reduce the need for surgery. Though with high heterogeneity and inconsistent sensitivity analysis result, the reduced surgery rate was similar to the results reported by Feng et al. that the surgical intervention rate was lower for AP patients who had received hemofiltration and peritoneal dialysis treatment than patients with routine treatment only [32]. Based on the above benefits brought by the continuous hemofiltration, the present study proposed continuous hemofiltration for SAP. Generally, the cost of a novel treatment strategy brings about the cost-effectiveness concern. Few studies have reported the cost-effectiveness analysis results of various hemofiltration modes for SAP, but the cost comparison to the routine treatment was not elucidated [33]. The present study confirms that continuous hemofiltration does not increase the expense burden considering the additional continuous hemofiltration therapy to the routine treatment. Moreover, the present meta-analysis conducted a subgroup analysis and showed that HVHF mode may improve the status of patients regarding the reduced APACHE II score at 72 h after the HVHF treatment. Although the levels of CRP, Cr, and Bun at the time point of 72 h were not significantly decreased, HVHF mode could still reduce the surgery rate and mortality rate of SAP patients effectively.

The previously reported durations of continuous hemofiltration were various, ranging from 24 h to 7 days [10, 15]. Some authors thought that the timing and duration of hemofiltration for SAP were important [34]. Thus, to explore the evidence explicating the duration of hemofiltration for effectively treating SAP would be of clinical significance. We collected these data of interest at different time points and analyzed the item of interest reported by more than three of the included studies at the same time point. In the present study, data involving time points such as Bun, Cr, ALT, CRP, and APACHE II were analyzed according to the same time point after the onset of treatment. The pooled results on Bun, Cr, ALT, CRP, and APACHE II indicated that continuous hemofiltration could effectively alleviate SAP as early as 72 hours after the onset of treatment. Moreover, in order to secure the efficacy and credibility of the pooled results, a study without baseline data was excluded [31, 35]. Besides, retrospective studies in which only SAP patients with complications could receive continuous hemofiltration treatment and constituted the hemofiltration group were not included in the present study, because this study design induced great bias. Lastly, the present study analyzed and concluded the data of abdominal pain relief time, surgery rate, and cost of hospitalization for the first time [8].

In the present study, there exist some limitations. Firstly, not all included studies are RCTs. Besides, most included RCTs are of a relatively low quality. Thus, these could affect the pooled results of the present study. Secondly, variations exist in the protocols regarding continuous hemofiltration therapy, patients, and treatment experiences among different medical centers. These variations might contribute to heterogeneity existing in some synthesized results. For example, high heterogeneity exists in the surgery rate and the sensitivity analysis result of this item was inconsistent. Thus, conclusions should be drawn with caution and justified. Thirdly, not all studies provided all outcomes of interest, which may affect the power of the analysis. Because data of interest under other hemofiltration modes were all reported by less than two of the included studies, the subgroup pooled analysis of these items regarding the other hemofiltration modes could not be conducted. Finally, the risk of bias always existed, even no severe publication bias was indicated by the above analysis.

## 5. Conclusion

The present study demonstrated that continuous hemofiltration therapy is safe and effective for SAP patients. Continuous hemofiltration therapy could effectively alleviate SAP as early as 72 hours after the onset of treatment, benefiting patients with lower levels of Bun, Cr, CRP, and APACHE II scores. Besides, continuous hemofiltration therapy could confer SAP patients with shorter abdominal pain relief time and lower mortality rate. RCTs in better design are still demanded to clarify these advantages.

## Figures and Tables

**Table 1:** Baseline characteristics and demographics of patients included.

References	Study type	Subtype of hemofiltration	Number		Age		Gender (male/female)		Etiological factors (gallstones/alcohol/hyperlipidemia/others)		Apache II score at admission		Amylase, *μ*/l		C-reactive protein, mg/L		Creatinine, *μ*mol/L		Score
			CH	CO	CH	CO	CH	CO	CH	CO	CH	CO	CH	CO	CH	CO	CH	CO	
[15]	Randomized controlled trial	CVVH	10	10	57.7 ± 19.4	60.7 ± 11.4	6/4	6/4	NR	NR	14.7 ± 3.3	13.1 ± 1.5	NR	NR	NR	NR	NR	NR	2^b^
[16]	Randomized controlled trial	CVVH	22	15	NR	NR	NR	NR	NR	NR	14.8 ± 4.5	14.6 ± 4.7	692 ± 273	680 ± 285	243 ± 18	242 ± 20	108 ± 21	109 ± 24	2^b^
[17]	Randomized controlled trial	CVVH	33	30	49.3 (19–81)	50.7 (19–79)	18/15	16/14	18/8/4/3	15/6/5/4	15.3 ± 5.7	14.9 ± 5.1	942.7 ± 815.1	1060.3 ± 987.3	NR	NR	NR	NR	2^b^
[18]	Randomized controlled trial	HVHF	30	30	44.1 ± 9.5	49.7 ± 17.0	21/9	18/12	NR	NR	15.3 ± 1.0	13.8 ± 3.6	NR	NR	NR	NR	235.3 ± 75.9	197.6 ± 61.2	2^b^
[19]	Prospective study	HVHF	32	29	53.0 ± 15.7	48.2 ± 12.5	12/20	10/19	NR/7/4/21	N/6/5/18	19.3 ± 4.7	19.4 ± 4.4	NR	NR	155.1 ± 49	153.4 ± 51.31	NR	NR	8^c^
[20]	Randomized controlled trial	Hemofiltration	32	32	52.31 ± 11.96	51.58 ± 12.64	21/11	23/9	NR	NR	20.37 ± 4.58	20.61 ± 3.58	NR	NR	29.81 ± 7.06	28.93 ± 7.24	126.30 ± 16.54	125.57 ± 15.81	2^b^
[21]	Randomized controlled trial	CVVDH	65	60	46.5 ± 10.4	45.6 ± 11.2	35/30	33/27	24/13/15/13	25/13/11/11	15.97 ± 1.63	16.66 ± 1.70	NR	NR	NR	NR	NR	NR	3^b^
[22]	Prospective study	HVHF	18	22	53.94 ± 16.46	50.55 ± 14.99	14/4	11/11	10/1/NR/7	10/0/NR/12	NR	NR	876.3 ± 178.0	619.9 ± 566.2	209.3 ± 171.4	227.8 ± 89.8	267 ± 48	263 ± 52	8^c^
[23]	Randomized controlled trial	Hemofiltration	46	46	38.87 ± 6.47	39.13 ± 6.56	24/22	22/24	7/16/20/3	9/14/18/5	18.75 ± 3.04	18.93 ± 3.16	NR	NR	174.28 ± 9.25	173.84 ± 9.48	NR	NR	2^b^

CH, continuous hemofiltration; CO, control; CVVH, continuous venovenous hemofiltration; CVVDH, continuous venovenous diahemofiltration; HVHF, high-volume hemofiltration. ^a^Significant difference. ^b^Randomized clinical trial (RCT), and the Jadad scale points. ^c^The Newcastle–Ottawa Scale (NOS) score.

**Table 2:** Summary of pooled results.

Pooled result	Statistical method	Number of studies	MD/OR	95% CI	*P* value	Heterogeneity	
						*P*	I^2^
APACHE II score at 24 h after treatment	Random	3	−2.41	−5.16, 0.34	0.09	0.0002^*∗∗*^	88
APACHE II score at 72 h after treatment	Random	5	−1.8	−3.15, −0.44	0.009^*∗∗*^	<0.00001^*∗∗*^	96
CRP at 72 h after treatment (mg/L)	Random	4	−1.56	−2.64, −0.47	0.005^*∗∗*^	<0.00001^*∗∗*^	91
ALT at 72 h after treatment (U/L)	Random	3	−0.35	−0.83, 0.13	0.16	0.10	57
Cr at 72 h after treatment (umol/L)	Random	4	−4.96	−7.77, −2.15	0.0005^*∗∗*^	<0.00001^*∗∗*^	98
Bun at 72 h after treatment (mmol/L)	Random	4	−3.63	−6.07, −1.20	0.003^*∗∗*^	<0.00001^*∗∗*^	97
Abdominal pain relief time (hours)	Random	3	−1.82	−2.93, −0.71	0.001^*∗∗*^	0.0006^*∗∗*^	86
Surgery rate	Random	3	0.15	0.03, 0.78	0.02^*∗*^	0.06	64
Mortality rate	Fixed	5	0.57	0.37, 0.85	0.007^*∗∗*^	0.98	0
Length of hospital stay after treatment (days)	Random	5	−0.51	−2.46, 1.44	0.61	<0.00001^*∗∗*^	97
Cost of hospitalization (10 000 RMB)	Random	3	−0.72	−1.64, 0.20	0.12	0.002^*∗∗*^	84

MD, mean difference; OR, odds ratio; CI, confidence interval. ^*∗*^Statistical difference, *P* < 0.05. ^*∗∗*^Statistical difference, *P* < 0.01.

**Table 3:** Subgroup pooled results for patients receiving continuous high-volume hemofiltration.

Pooled result	Statistical method	Number of studies	MD/OR	95% CI	*P* value	Heterogeneity	
						*P*	I^2^ (%)
APACHE II score at 72 h after treatment	Fixed	3	−0.97	−1.30, −0.64	<0.00001^*∗∗*^	0.24	30
CRP at 72 h after treatment (mg/L)	Random	2	−1.01	−2.22, 0.21	0.11	0.005^*∗∗*^	88
ALT at 72 h after treatment (U/L)	Fixed	2	−0.56	−0.96, −0.15	0.007^*∗∗*^	0.15	51
Cr at 72 h after treatment (umol/L)	Random	2	−1.75	−4.10, 0.60	0.14	<0.00001^*∗∗*^	95
Bun at 72 h after treatment (mmol/L)	Random	2	−1.32	−2.72, 0.08	0.06	0.003^*∗∗*^	89
Surgery rate	Random	2	0.17	0.07, 0.42	0.0001^*∗∗*^	0.02^*∗*^	82
Mortality rate	Fixed	3	0.55	0.33, 0.92	0.02^*∗*^	0.82	0
Length of hospital stay after treatment (days)	Random	2	1.24	−3.34, 5.82	0.59	<0.00001^*∗∗*^	99

MD, mean difference; OR, odds ratio; CI, confidence interval. ^*∗*^Statistical difference, *P* < 0.05. ^*∗∗*^Statistical difference, *P* < 0.01.

## Data Availability

The data supporting this meta-analysis are from previously reported studies and datasets, which have been cited. All data generated or analyzed during this study are included in this published article. The processed data are available from the corresponding author upon reasonable request.

## Abbreviations

SAP: Severe acute pancreatitis
SIRS: Systemic inflammatory response syndrome
MODS: Multiple organ dysfunction syndrome
CRRT: Continuous renal replacement therapy
NOS: Newcastle–Ottawa scale
RCTs: Randomized clinical trials
MDs: Mean differences
CIs: Confidence intervals
ORs: Odds ratios
APACHE II: Acute Physiology and Chronic Health Evaluation II
AKI: Acute kidney injury
CARS: Compensatory anti-inflammatory response syndrome
Bun: Blood urea nitrogen
Cr: Creatinine
ALT: Alanine transaminase
CRP: C-reactive protein
CVVH: Continuous venovenous hemofiltration.
